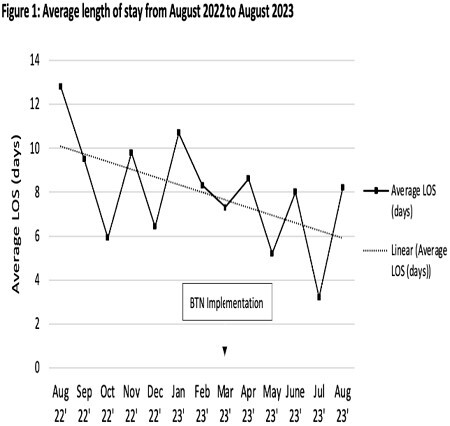# 551 Improving Outpatient Access and Reducing Length of Stay Through a Burn Treatment Nurse Pilot Program

**DOI:** 10.1093/jbcr/irae036.185

**Published:** 2024-04-17

**Authors:** tracey tonkinson, Taylor Powell, Mona Chambers, Charles Sandidge, Caitlin M Orton, Tam N Pham

**Affiliations:** Harborview Medical Center, Seattle, WA; UW Medicine - Harborview Burn Center, Seattle, WA; University of Washington, Lynnwood, WA; UW Medicine Regional Burn Center, Harborview Medical Center, Seattle, WA; University of Washington, Harborview Burn Centre, Seattle, WA; Harborview Medical Center, Seattle, WA; UW Medicine - Harborview Burn Center, Seattle, WA; University of Washington, Lynnwood, WA; UW Medicine Regional Burn Center, Harborview Medical Center, Seattle, WA; University of Washington, Harborview Burn Centre, Seattle, WA; Harborview Medical Center, Seattle, WA; UW Medicine - Harborview Burn Center, Seattle, WA; University of Washington, Lynnwood, WA; UW Medicine Regional Burn Center, Harborview Medical Center, Seattle, WA; University of Washington, Harborview Burn Centre, Seattle, WA; Harborview Medical Center, Seattle, WA; UW Medicine - Harborview Burn Center, Seattle, WA; University of Washington, Lynnwood, WA; UW Medicine Regional Burn Center, Harborview Medical Center, Seattle, WA; University of Washington, Harborview Burn Centre, Seattle, WA; Harborview Medical Center, Seattle, WA; UW Medicine - Harborview Burn Center, Seattle, WA; University of Washington, Lynnwood, WA; UW Medicine Regional Burn Center, Harborview Medical Center, Seattle, WA; University of Washington, Harborview Burn Centre, Seattle, WA; Harborview Medical Center, Seattle, WA; UW Medicine - Harborview Burn Center, Seattle, WA; University of Washington, Lynnwood, WA; UW Medicine Regional Burn Center, Harborview Medical Center, Seattle, WA; University of Washington, Harborview Burn Centre, Seattle, WA

## Abstract

**Introduction:**

To address a growing volume of both external referrals and follow up needs within a regional burn center, the outpatient burn clinic tested the feasibility and potential benefits of a Burn Treatment Nurse (BTN) Program. This pilot utilized a full time RN position to perform wound care for inpatient and outpatient anesthesia assisted procedures (AAP’s), and to independently evaluate acute burn referrals and post discharge patients with a quick turnaround time. This analysis reports on the first 6 months results by service level utilization and performance of initiative.

**Methods:**

The BTN program was implemented following a collaborative effort among the outpatient clinic, the burn service line and hospital administration. As part of the BTN QI tracking, we prospectively collected newly generated clinic revenue, number of independent RN visits, length of inpatient length of stay (LOS) on acute care and collected feedback from provider stakeholders.

**Results:**

At the conclusion of the first 6 months, the BTN program has allowed for an additional 357 patient visits. It generated an additional revenue of $286,129, a 19% increase in revenue for the burn clinic. The BTN also contributed to an overall decrease in LOS on acute care. Pre implementation, mean LOS was 9.2 days, after implementation mean LOS was 6.8 days (Figure 1). Not only did BTN provide positive outcomes for the clinic and hospital, anesthesia and nursing providers expressed an overall feeling of success and strong support for the continuation of the BTN program.

**Conclusions:**

The BTN capability has expanded critical access for those needing expedited outpatient care. This innovative program has improved outpatient access and contributed to decreased inpatient LOS for our regional burn center.

**Applicability of Research to Practice:**

Burn centers with a growing outpatient volume should consider implementing a nurse with flexible inpatient/outpatient roles to improve both outpatient and inpatient access efficiency.